# Ball versus Locator^®^ Attachments: A Retrospective Study on Prosthetic Maintenance and Effect on Oral-Health-Related Quality of Life

**DOI:** 10.3390/ma14041051

**Published:** 2021-02-23

**Authors:** Silvia Brandt, Hans-Christoph Lauer, Michael Fehrenz, Jan-Frederik Güth, Georgios Romanos, Anna Winter

**Affiliations:** 1 Department of Prosthodontics, Goethe University Frankfurt, Theodor-Stern-Kai 7, 60596 Frankfurt am Main, Germany; gueth@med.uni-frankfurt.de; 2 Private Practice B2V Dr. Brandt and Colleagues, 61118 Bad Vilbel, Germany; h.c.lauer@freenet.de; 3 Clinic for Oral and Maxillofacial Surgery, Saarland University Hospital, Kirrberger Str. 100, 66424 Homburg, Germany; mf21@me.com; 4 Department of Periodontology, School of Dental Medicine, Stony Brook University, Rockland Hall 106, Stony Brook, NY 11794-8700, USA; Georgios.Romanos@stonybrook.edu; 5 Department of Oral Surgery and Implant, Goethe University Frankfurt, Theodor-Stern-Kai 7, 60596 Frankfurt am Main, Germany; 6 Department of Prosthodontics, Julius Maximilian University Würzburg, Pleicherwall 2, 97070 Würzburg, Germany; Winter_A3@ukw.de

**Keywords:** attachment, ball, locator, overdenture, OHRQoL, OHIP-G14

## Abstract

Locator^®^ and ball attachments are well-established systems to attach overdentures to two inter-foraminal implants. This study aimed to evaluate differences between the two systems regarding prosthetic maintenance and patients’ oral-health-related quality of life (OHRQoL). Dental records of patients with a mandibular implant-retained overdenture were retrospectively analyzed. Prosthetic maintenance measures involving the denture suprastructure and attachment matrix and patrix were analyzed. Furthermore, the Oral Health Impact Profile-G14 (OHIP-G14) was used to evaluate OHRQoL. Results were analyzed by means of Kaplan–Meier analysis and Student’s *t*- and log-rank tests. The records of 122 patients were evaluated. Kaplan–Meier survival analysis revealed a significant difference between ball attachments (Group B; *n* patients = 47) and Locator^®^ attachments (Group L; *n* patients = 75) regarding the occurrence of denture fractures (*p* < 0.001) and events affecting the matrix (*p* = 0.028) and patrix (*p* = 0.030). Group L had a significantly lower total OHIP-G14 score than Group B (*p* = 0.002). The most common maintenance events were matrix-related and denture relining for both attachment systems. Group B required more maintenance measures than Group L. Moreover, patients in Group L had better OHRQoL than patients in Group B.

## 1. Introduction

The number of edentulous patients is declining; nonetheless, treatments of edentulous jaws remain an essential part of day-to-day dental practice. Among edentulous patients, in particular, the mandible exhibits severe atrophy of the alveolar ridge, which can result in inadequate denture retention and restricted denture function, and thus in an associated reduction in patient satisfaction [1]. To overcome this problem, dental implants can be inserted to enhance stable seating of the denture. A removable denture can subsequently be attached to the mandible in a comparable minimally invasive way by means of two interforaminal implants [2,3].

The aims of using an implant-retained denture are to improve masticatory efficiency, masticatory force, and patients’ oral-health-related quality of life (OHRQoL) [4,5]. The choice of attachment type and the retention it provides can affect the improvement being targeted, as well as the survival rates of the implants and dentures [6,7].

Various attachment systems—each with its own particular advantages and disadvantages—are available for the retention of removable dentures. In this context, double-crown-retained systems deliver good retention and high patient satisfaction; however, they are highly technique-sensitive and very costly [8]. A magnet or bar attachment can also be used, but these systems have been less clinically successful [9,10]. By contrast, ball-retained dentures have yielded good clinical results, probably because their retention inserts can be replaced, thus improving the denture’s long-term prognosis. Retention inserts can also be replaced and adjusted in the Locator^®^ system. The attachments in both systems are small in height, which has a positive effect on the transmission of stress to peri-implant areas during functional movements [11,12]. Based on the results of previous studies, these two similar attachment systems can therefore be characterized as well-established therapeutic options that are associated with good clinical results and high patient satisfaction [13,14,15,16,17,18,19].

In this context, the measures of patient satisfaction and OHRQoL—which is affected by patient satisfaction—can also be used to evaluate treatment success. The Oral Health Impact Profile (OHIP), which is available in several different versions and languages, is a suitable means to assess OHRQoL during dental treatment [20]. The OHIP-G14 contains 14 questions, and the German-language version constitutes a validated tool for measuring OHRQoL [21].

To make it easier for practitioners to decide between the two types of attachment, the aim of this study was to investigate and quantify possible differences in terms of prosthetic maintenance and OHRQoL.

The null hypothesis was that dentures with Locator^®^ and ball attachments would not differ with regard to prosthetic maintenance and OHRQoL.

## 2. Materials and Methods

This retrospective study analyzed the prosthetic maintenance of patients with a mandibular implant-supported overdenture. Patients from three private dental practices who received an overdenture to restore an edentulous mandible between January 2004 and October 2018 were included in the study. Patients had to meet the inclusion criterion of an overdenture on two interforaminal implants retained by means of ball or Locator attachments. To be included in the study, patients were also required to regularly attend maintenance appointments for a minimum period of 18 months. Patients with incomplete records or alcohol/drug abuse problems were excluded. The patients’ final appointment was defined by the endpoint of the observation period. The responsible Ethics Committee at the Medical Council of Saarland, Germany, approved the study design (approval number: 153/18; 22 October 2018).

For the collection of data, the prosthetic maintenance measures carried out during the maintenance appointments were analyzed. A distinction was made among superstructure-related maintenance measures (denture fracture, remaking, relining), and repair to matrix and patrix components. Matrix-related events were divided into three categories: “Re-adjusted,” “re-attached,” or “replaced”. Patrix-related events were categorized as “re-attached” or “replaced”.

In addition, the German version of the OHIP-G14 was used to evaluate OHRQoL in relation to each type of attachment. This questionnaire comprises 14 items related to seven domains: Functional limitation, physical pain, psychological discomfort, physical disability, psychological disability, social disability, and handicap [20]. These domains are assessed by means of questions such as, “Have you found it uncomfortable to eat any foods because of problems with your teeth, mouth or dentures?” or “Have you had to interrupt meals because of problems with your teeth, mouth or dentures?” [20,21]. All patients completed the OHIP-G14 questionnaire once at the start of the study. Patients were asked to report how often the respective OHIP parameter had occurred during the past month. Standardized response categories based on a five-point Likert scale were used (0 = never, 1 = rarely, 2 = occasionally, 3 = fairly often, and 4 = often) [22]. After the assessment, a total OHIP score was calculated. A lower OHIP score indicates better oral health.

IBM SPSS Statistics 25 (SPSS Inc.; IBM Company, Chicago, IL, USA) was used for statistical analysis. The Kolmogorov–Smirnov test was used to test for a normal distribution, and then analysis by means of the Mann–Whitney U test, chi-squared test, and Fisher’s exact test was carried out. Student’s *t*- and log-rank tests were used to compare the two treatment groups. Kaplan–Meier analyses were also conducted. The significance level was set at *p* = 0.05.

## 3. Results

Notably, 122 patients (men: 52, women: 70, mean age: 63 ± 11.5 years) who received an overdenture on two interforaminal implants retained either by ball (Group B; *n* of patients = 47) or Locator^®^ (Group L; *n* of patients = 75) attachments met the inclusion criteria and were enrolled. The mean observation period was 116 ± 51.6 months for Group B and 88 ± 52.5 months for Group L.

The following implant systems were used to retain the attachments being investigated: Straumann (32%; Basel, Switzerland), Astra Tech (26%; Dentsply-Sirona; Charlotte, NC, USA), Bredent (23%; Senden, Germany), Zimmer-Biomet (12%; Munich, Germany), and ZEST (7%; Carlsbad, CA, USA). The mean implant length was 12 mm and the mean diameter was 3.4 mm. During the entire observation period, two implants were removed in total (both in Group B).

Table 1 shows the number of maintenance measures per denture. In both groups, the most commonly required maintenance measures were matrix-related, at 6.1 ± 4.9 (Group B) and 3.0 ± 3.5 (Group L) events per denture. Table 2 provides an overview of the general incidence of maintenance, stratified by type of maintenance measure. Within 12 months, only a small number of patrix-related events (≤1.4 ± 1.3%), denture refabrication (≤1.4 ± 1.3%), and denture fracture (≤4.3 ± 2.9%) occurred in both groups. The need for denture relinings appeared in 36.2 ± 7% (Group B) and 33.5 ± 5.5% (Group L), and matrix-related events in 40.2 ± 7.2% (Group B) and 28.3 ± 5.2% (Group L).

**Figure 1:**
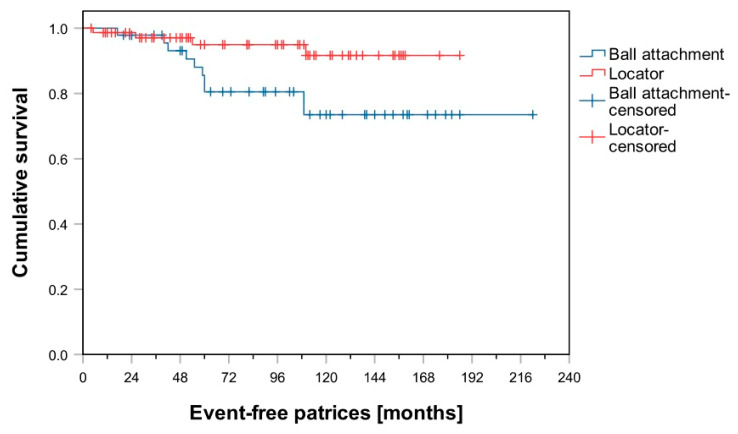
Kaplan–Meier survival curves for ball and Locator attachments regarding patrix-related events.

**Figure 2:**
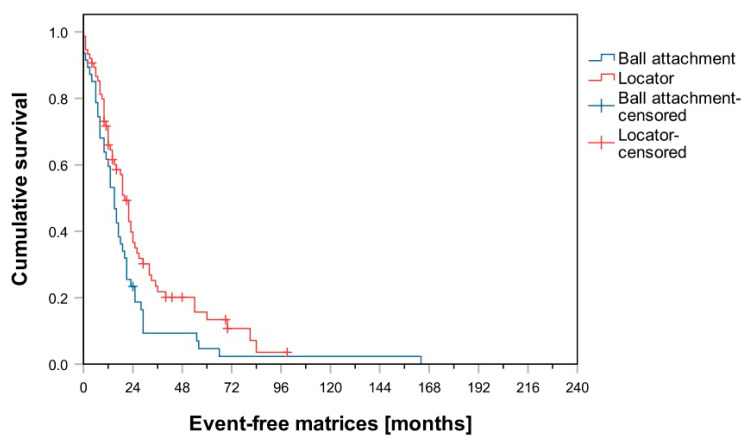
Kaplan–Meier survival curves for ball and Locator attachments regarding matrix-related events.

**Figure 3:**
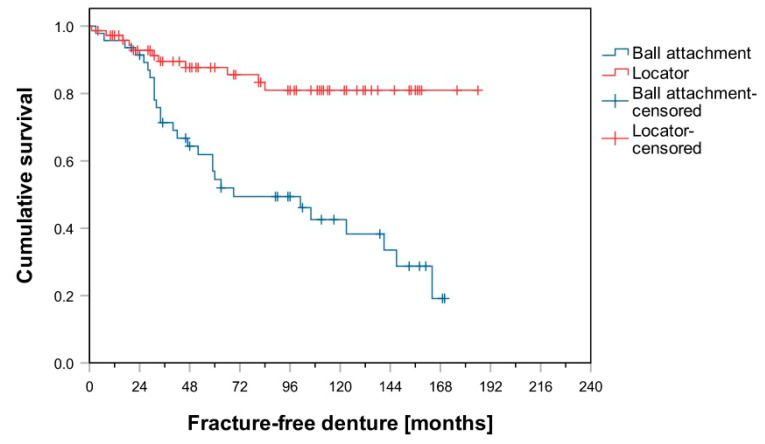
Kaplan–Meier survival curves for “denture fracture”: Ball versus Locator attachments.

After an observation period of 60 months, 86% of patients in Group B had not been affected by any “patrix-related events”. In Group L, this figure was 95%. Comparison of the Kaplan–Meier analysis shows a significant difference in favor of Group L with regard to “patrix-related events” (log-rank test, *p* = 0.030; Figure 1). Group L was also significantly superior in terms of “matrix-related events” (log-rank test, *p* = 0.028; Figure 2) and “denture fracture” (log-rank test, *p* < 0.001; Figure 3). No significant difference was observed between groups L and B regarding the events “re-lining” and “re-fabrication”. In total, 36 dentures had to be refabricated (Group B: *n* = 19; Group L: *n* = 17).

The OHIP-G14 questionnaire was filled out by 78 patients after the completion of prosthetic treatment (Group B: 27, Group L: 51). Analysis yielded a total OHIP score (mean ± SD) of 10.4 ± 4.45 points for Group L and 13.9 ± 5.16 points for Group B, signifying a significant difference between the two groups (*t*-test, *p* = 0.002).

## 4. Discussion

The aim of this study was to evaluate possible differences between Locator^®^ and ball attachments in implant-retained mandibular overdentures in terms of prosthetic maintenance. The results revealed that the two attachment systems did differ with regard to prosthetic maintenance and the effect on OHRQoL. The null hypothesis could therefore be rejected.

Analysis of the evaluated maintenance measures demonstrated an increasing number of necessary maintenance associated with increasing observation time. In general, a higher need for maintenance occurred in dentures retained by ball attachments, which negatively affects their survival time compared to prostheses in Group L.

Matrix-related events were the most common maintenance events in both groups. In this study, the matrices of Group L had to be adjusted less often than those of Group B. Kaplan–Meier survival analysis showed that this difference was statistically significant. Within the prosthetic maintenance measures, 59.8 ± 7.2% (Group B) and 71.7 ± 5.2% (Group L) of dentures remained unaffected by matrix-related events after one year. After just 24 months, a further substantial decrease in these values had occurred. This suggests that the need for matrix-related maintenance is high and occurs shortly after the completion of treatment. In general, the readjustment of matrices represented the main reason for “matrix-related events”. 

In this context, matrices often need to be adjusted due to a loss of retention, which is also mentioned in the literature as a common reason for prosthetic maintenance [23]. With regard to Locator^®^-retained overdentures, Elsyad et al. showed a high initial retention for various matrices, and only a small loss of retention after cyclic loading [24]. To ensure long-lasting retentive stability, it is therefore advised to precisely adjust the retention of the prosthesis at the point of its incorporation [25]. Sultana et al. found that retention was initially higher for Locator^®^-retained dentures than for ball attachments [26], which is consistent with the results of the present study. After artificial ageing by means of cyclic loading, a loss of retention was observed for both types of attachment; however, this was more pronounced for Locator than for ball attachments [26]. Taking the loss of retention as the most common reason for maintenance, this is not supported by the present study. One influencing factor on the premature loss of retention might be the angulation of the implants. A relationship between inter-implant angulation and a negative effect on retention behavior has been described in relation to both Locator and ball attachments [26,27]. In addition, and as a limitation of this study, it should be mentioned that the matrix structure in Group B was not uniform. Both one-piece and two-piece matrix systems were used. Thus, patrix–matrix retention was achieved using a lamella, a ring spring, or resin inserts in the matrix. These differences could have been an influencing factor for the ratio of adjustment needs observed in Group B.

Relining was the second-most common type of prosthetic maintenance required. Within 24 months after incorporation, approximately 55% (group B) and 46% (group L) of dentures in both groups required relining. Considering 5 years post-incorporation, about 72% of dentures underwent relining. No significant difference was observed between the two groups.

By contrast, a significant difference in favor of Group L was observed in terms of the time until first denture fracture. This is consistent with previous studies by Vahidi et al. and Fernadez-Estevan et al., which also describe the relining and repair of prosthesis fracture as common prosthetic maintenance measures [28,29]. A possible cause for this might be the resilient support of the overdentures investigated [30]. In this context, regular relining ensures a good fit between the denture base and foundation area, which might reduce the probability of fracture, having a positive effect on patient satisfaction and the functionality and long-term stability of the denture [31]. If the fit between the denture and foundation area is poor, or if areas of the denture are subjected to severe stresses, then a deformity in the base of the complete denture can result in denture fractures [32]. Elsyad et al. found that denture deformations in the area above the implants were significantly worse in ball-retained overdentures than in the Locator^®^-retained alternative [33]. This less distinct point of weakest resistance could be one reason why denture fractures were less common in Group L. Based on the results of the present and previously published studies, reinforcement of the denture base by metal structures can be recommended, particularly if ball attachments are used [33].

Although only a small number of patrix-related events occurred, Kaplan–Meier survival analysis showed that Group L was significantly superior in this regard. In this context, the time-to-first event was significantly better for the dentures in Group L than for those in Group B. The events “re-attachment” and “replacement of matrices” were observed, which can be caused by abutments becoming loose or worn. In their in vitro study, Aroso et al. observed no wear on metal abutments after cyclic loading [34]. This is consistent with the generally low incidence of this event in the present study. However, wear is also affected by patrix angulation, which was not considered in the current study [34]. Furthermore, the abutment-surface finish also has an effect on wear. Yabul et al. showed a significant relationship between the extent of wear and the matrix and patrix materials used in ball attachments [35], which might explain the higher incidence of this event in Group B.

In addition to analyzing patients’ prosthetic maintenance measures, the present study also investigated OHRQoL. Analysis of the results of the OHIP-G14 questionnaire revealed that the study patients’ OHRQoL was less impaired than that of most patients who are edentulous or wear complete dentures [36]. A possible explanation for the findings could be that wearing implant-supported prostheses results in an increase in oral function. In general, an improvement in speech quality after prosthetic rehabilitation was observed by Knipfer et al. [37]. Furthermore, Müller et al. described a positive effect on chewing efficiency for implant-supported overdentures [38].

Furthermore, OHRQoL was affected by the attachment system used. The results indicate that OHRQoL was significantly higher in Group L, as signified by Group B’s significantly higher OHIP scores. Nevertheless, the average scores from the OHIP evaluations were higher in this study than in previous studies [16,39]. It should be noted, however, that a baseline evaluation was not performed in the present study, which is of relevance regarding OHIP score categorization [39]. Therefore, the assessment before treatment is missing in both groups. Thus, the change in OHIP score and the impact of treatment on OHIP score could not be investigated, which must be taken into account when interpreting the results. Furthermore, Deeb et al. demonstrated that OHRQoL among patients with removable dentures is also affected by socio-economic, demographic, and anamnestic parameters [40]. The effect of these parameters was not assessed in the present study, which constitutes a further limitation regarding the assessment of OHRQoL.

Another is the time of OHRQoL assessment. All patients answered the OHIP questionnaire once at the start of the retrospective study, not at a defined time after receiving their dentures. The length of time between completion of treatment and assessment of OHRQoL therefore differed among the patients, which could also have affected the outcome of the study [16].

In their study of a similar design, Kuoppala et al. also assessed OHRQoL related to implant-supported overdentures in a retrospective manner [41]. However, unlike in the present study, Kuoppala et al. analyzed demographic factors.

Furthermore, a possible relationship between OHRQoL and maintenance measures required has been described previously [16]. This agrees with the results of the present study, whereby Group L’s lower maintenance requirements might have had a positive effect on their OHRQoL.

When considering the results of this study, one limitation to consider is that the patients were treated by different practitioners. As a result, possible practitioner-dependent influencing factors need to be considered. On the other hand, multiple practitioners minimize the influence of possible bias regarding clinical decision making by one practitioner. Further, this approach has been used in previous studies and corresponds to common practice [42,43]. To minimize any influence from the respective practitioner, a standardized treatment procedure was selected for the fabrication of both types of denture. As a result, the study only included dentures that had been fabricated in accordance with the relevant manufacturer specifications.

Another limitation of the study is its retrospective design, which was based on the records of different dentists [43]. Although a standardized procedure was used, the possibility of inconsistent documentation cannot be ruled out, which might have affected the study results. Here, too, a standardized procedure was used to try to ensure that the records of the different dentists were as comparable as possible. 

Finally, another limitation is that groups L and B differed in size and observation time. However, because the group sizes depended on the patients’ choice of preferred attachment system, it was not possible to control this parameter. The influence of this parameter was limited as far as possible by the method of data analysis, e.g., reporting results by year. Nevertheless, to reduce the effects of patient-related factors, further prospective studies with equal group size and observation periods, as well as an interindividual comparison of both attachment systems, are recommended.

## 5. Conclusions

Within the limitations of the present study, the following conclusions can be drawn:

- Fewer maintenance measures were required for implant-retained overdentures retained by Locator^®^ attachments than for those retained by ball attachments.
- Denture fracture occurred more frequently when ball attachments were applied.
- In both groups, the most common maintenance measures were matrix-related events, re-lining, and denture fractures.
- OHRQoL was significantly higher among patients in Group L than among patients in Group B.

## Figures and Tables

**Table 1:** Incidence of maintenance measures per denture (± SD), showing a decreasing trend.

Maintenance Measure	Group B	Group L
Matrix-related events per denture	-	-
Readjusted	5.1 ± 4.1	2.8 ± 3.1
Reattached	0.3 ± 0.5	0 ± 0.2
Replaced	0.8 ± 0.8	0.1 ± 0.5
Total	6.1 ± 4.9	3.0 ± 3.5
Denture relining	2.28 ± 1.75	1.23 ± 1.11
Denture fracture	1.09 ± 1.21	0.21 ± 0.58
Patrix-related events per denture	-	-
Reattached	0.1 ± 0.4	0.1 ± 0.2
Replaced	0.1 ± 0.3	0 ± 0.2
Total	0.3 ± 0.6	0.1 ± 0.4
Denture re-fabrication	0.57 ± 0.77	0.25 ± 0.50
Total number of documented events	10.28 ± 7.66	4.75 ± 4.84

Ball attachments (Group B)/Locator attachments (Group L).

**Table 2:** Incidence of maintenance in % (±SD), stratified by group (B/L) and maintenance measure.

Maintenance Measure	Within 24 Months		Within 60 Months	
	Group B	Group L	Group B	Group L
Patrix-related events	2.1 ± 2.1	1.4 ± 1.3	19.5 ± 6.2	5.1 ± 2.9
Denture refabrication	0	2.8 ± 2	12.7 ± 5.3	11.9 ± 4.3
Denture fracture	8.6 ± 4.1	7.2 ± 3.1	45.5 ± 7.6	12.3 ± 4.1
Denture relining	55.7 ± 7.3	46.1 ± 6	72 ± 6.7	72.9 ± 5.8
Matrix-related events	76.6 ± 6.2	60.2 ± 6	95.3 ± 3.2	86.6 ± 4.6

## Data Availability

The data presented in this study are available on request from the corresponding author.

## References

[1] Polzer I., Schimmel M., Muller F., Biffar R. (2010). Edentulism as part of the general health problems of elderly adults. Int. Dent. J..

[2] Kern J.S., Kern T., Wolfart S., Heussen N. (2016). A systematic review and meta-analysis of removable and fixed implant-supported prostheses in edentulous jaws: Post-loading implant loss. Clin. Oral. Implant. Res..

[3] Thomason J.M., Kelly S.A., Bendkowski A., Ellis J.S. (2012). Two implant retained overdentures--a review of the literature supporting the McGill and York consensus statements. J. Dent..

[4] Sharma A.J., Nagrath R., Lahori M. (2017). A comparative evaluation of chewing efficiency, masticatory bite force, and patient satisfaction between conventional denture and implant-supported mandibular overdenture: An in vivo study. J. Indian Prosthodont Soc..

[5] Zhang L., Lyu C., Shang Z., Niu A., Liang X. (2017). Quality of life of implant-supported overdenture and conventional complete denture in restoring the edentulous mandible: A systematic review. Implant. Dent..

[6] Elsyad M.A., Shawky A.F. (2017). Masticatory function with ball and resilient telescopic anchors of mandibular implant-retained overdentures: A crossover study. Quintessence Int..

[7] Elsyad M.A., Hegazy S.A., Hammouda N.I., Al-Tonbary G.Y., Habib A.A. (2014). Chewing efficiency and electromyographic activity of masseter muscle with three designs of implant-supported mandibular overdentures. A cross-over study. Clin. Oral. Implants Res..

[8] Brandt S., Brandt J., Ketabi A.R., Lauer H.C., Kunzmann A. (2019). Locator^®^ versus ceramic/electroplated double-crown attachments: A prospective study on the intraindividual comparison of implant-supported mandibular prostheses. Clin. Oral. Investig..

[9] Naert I., Alsaadi G., Quirynen M. (2004). Prosthetic aspects and patient satisfaction with two-implant-retained mandibular overdentures: A 10-year randomized clinical study. Int. J. Prosthodont.

[10] Gotfredsen K., Holm B. (2000). Implant-supported mandibular overdentures retained with ball or bar attachments: A randomized prospective 5-year study. Int. J. Prosthodont.

[11] Khurana N., Rodrigues S., Shenoy S., Saldanha S., Pai U., Shetty T., Mahesh M., Hegde P. (2019). A Comparative Evaluation of Stress Distribution with Two Attachment Systems of Varying Heights in a Mandibular Implant-Supported Overdenture: A Three-Dimensional Finite Element Analysis. J. Prosthodont.

[12] Dashti M.H., Atashrazm P., Emadi M.I., Mishaeel S., Banava S. (2013). The effects of two attachment types on the stresses introduced to the mandibular residual ridge: A 3D finite element analysis. Quintessence Int.

[13] Krennmair G., Seemann R., Weinlander M., Piehslinger E. (2011). Comparison of ball and telescopic crown attachments in implant-retained mandibular overdentures: A 5-year prospective study. Int. J. Oral. Maxillofac. Implants.

[14] Vere J., Hall D., Patel R., Wragg P. (2012). Prosthodontic maintenance requirements of implant-retained overdentures using the Locator attachment system. Int. J. Prosthodont.

[15] Liu W., Zhang X., Qing H., Wang J. (2019). Effect of LOCATOR attachments with different retentive forces on the stability of 2-implant-retained mandibular overdenture. J. Prosthet. Dent..

[16] Matthys C., Vervaeke S., Besseler J., Doornewaard R., Dierens M., De Bruyn H. (2019). Five years follow-up of mandibular 2-implant overdentures on Locator or ball abutments: Implant results, patient-related outcome, and prosthetic maintenance. Clin. Implant. Dent. Relat Res..

[17] Zweers J., van Doornik A., Hogendorf E.A., Quirynen M., Van der Weijden G.A. (2015). Clinical and radiographic evaluation of narrow- vs. regular-diameter dental implants: A 3-year follow-up. A retrospective study. Clin. Oral. Implants Res..

[18] Turk P.E., Geckili O., Turk Y., Gunay V., Bilgin T. (2014). In vitro comparison of the retentive properties of ball and Locator attachments for implant overdentures. Int. J. Oral. Maxillofac. Implants.

[19] Krennmair G., Seemann R., Fazekas A., Ewers R., Piehslinger E. (2012). Patient preference and satisfaction with implant-supported mandibular overdentures retained with ball or Locator attachments: A crossover clinical trial. Int. J. Oral. Maxillofac. Implants.

[20] Slade G.D. (1997). Derivation and validation of a short-form oral health impact profile. Community Dent. Oral. Epidemiol..

[21] John M.T., Miglioretti D.L., LeResche L., Koepsell T.D., Hujoel P., Micheelis W. (2006). German short forms of the Oral Health Impact Profile. Community Dent. Oral. Epidemiol..

[22] Sato N., Koyama S., Mito T., Izumita K., Ishiko R., Yamauchi K., Miyashita H., Ogawa T., Kosaka M., Takahashi T. (2019). Changes in oral health-related quality of life after oral rehabilitation with dental implants in patients following mandibular tumor resection. J. Oral. Sci..

[23] Kleis W.K., Kammerer P.W., Hartmann S., Al-Nawas B., Wagner W. (2010). A comparison of three different attachment systems for mandibular two-implant overdentures: One-year report. Clin. Implant. Dent. Relat. Res..

[24] ELsyad M.A., Dayekh M.A., Khalifa A.K. (2019). Locator Versus Bar Attachment Effect on the Retention and Stability of Implant-Retained Maxillary Overdenture: An In Vitro Study. J. Prosthodont.

[25] Evtimovska E., Masri R., Driscoll C.F., Romberg E. (2009). The change in retentive values of Locator attachments and hader clips over time. J. Prosthodont.

[26] Sultana N., Bartlett D.W., Suleiman M. (2017). Retention of implant-supported overdentures at different implant angulations: Comparing Locator and ball attachments. Clin. Oral. Implants Res..

[27] Teimoori H., Shayegh S.S., Zavaree M.A., Hakimaneh S.M., Khodadad F., Shidfar S., Baghani M.T. (2018). Effects of Excessive Implant Angulation on Retention of Two Types of Overdenture Attachments during Cyclic Loading. J. Contemp. Dent. Pract..

[28] Vahidi F., Pinto-Sinai G. (2015). Complications associated with implant-retained removable prostheses. Dent. Clin. North. Am..

[29] Fernandez-Estevan L., Montero J., Selva Otaolaurruchi E.J., Sola Ruiz M.F. (2018). Interventions to Maintain Locator-Retained Mandibular Overdentures on Both External Hex and Internal Connection Implants: A Retrospective Study. Int. J. Oral. Maxillofac. Implants.

[30] Nguyen C.T., Masri R., Driscoll C.F., Romberg E. (2010). The effect of denture cleansing solutions on the retention of pink Locator attachments: An in vitro study. J. Prosthodont.

[31] Fouda S.M., Al-Attar M.S., Virtanen J.I., Raustia A. (2014). Effect of Patient’s Personality on Satisfaction with Their Present Complete Denture and after Increasing the Occlusal Vertical Dimension: A Study of Edentulous Egyptian Patients. Int. J. Dent..

[32] Prombonas A.E., Vlissidis D.S. (2006). Comparison of the midline stress fields in maxillary and mandibular complete dentures: A pilot study. J. Prosthet. Dent..

[33] ELsyad M.A., Errabti H.M., Mustafa A.Z. (2016). Mandibular Denture Base Deformation with Locator and Ball Attachments of Implant-Retained Overdentures. J. Prosthodont.

[34] Aroso C., Silva A.S., Ustrell R., Mendes J.M., Braga A.C., Berastegui E., Escuin T. (2016). Effect of abutment angulation in the retention and durability of three overdenture attachment systems: An in vitro study. J. Adv. Prosthodont.

[35] Yabul A., Dayan C., Geckili O., Bilhan H., Tuncer N. (2018). Evaluation of volumetric wear of abutments on the retention loss of ball attachment systems in implant-retained overdentures: An in vitro study. Clin. Implant. Dent. Relat. Res..

[36] John M.T., Micheelis W., Biffar R. (2004). Normwerte mundgesundheitsbezogener Lebensqualität für Kurzversionen des Oral Health Impact Profile [Reference values in oral health-related quality of life for the abbreviated version of the Oral Health Impact Profile]. Schweiz Monatsschr. Zahnmed.

[37] Knipfer C., Bocklet T., Noeth E., Schuster M., Sokol B., Eitner S., Nkenke E., Stelzle F. (2012). Speech intelligibility enhancement through maxillary dental rehabilitation with telescopic prostheses and complete dentures: A prospective study using automatic, computer-based speech analysis. Int. J. Prosthodont.

[38] Müller F., Hernandez M., Grütter L., Aracil-Kessler L., Weingart D., Schimmel M. (2012). Masseter muscle thickness, chewing efficiency and bite force in edentulous patients with fixed and removable implant-supported prostheses: A cross-sectional multicenter study. Clin Oral Implants Res..

[39] Khalid T., Yunus N., Ibrahim N., Elkezza A., Masood M. (2017). Patient-reported outcome and its association with attachment type and bone volume in mandibular implant overdenture. Clin. Oral. Implants Res..

[40] Deeb M.A., Abduljabbar T., Vohra F., Zafar M.S., Hussain M. (2020). Assessment of factors influencing oral health-related quality of life (OHRQoL) of patients with removable dental prosthesis. Pak. J. Med. Sci..

[41] Kuoppala R., Näpänkangas R., Raustia A. (2013). Quality of Life of Patients Treated With Implant-Supported Mandibular Overdentures Evaluated With the Oral Health Impact Profile (OHIP-14): A Survey of 58 Patients. J. Oral. Maxillofac. Res..

[42] Reich S., Schierz O. (2013). Chair-side generated posterior lithium disilicate crowns after 4 years. Clin. Oral Investig..

[43] Guédat C., Nagy U., Schimmel M., Muller F., Srinivasan M. (2018). Clinical performance of LOCATOR^®^ attachments: A retrospective study with 1–8 years of follow-up. Clin. Exp. Dent. Res..

